# The *paf* gene product modulates asexual development in *Penicillium chrysogenum*

**DOI:** 10.1002/jobm.201000321

**Published:** 2011-06

**Authors:** Nikoletta Hegedüs, Claudia Sigl, Ivo Zadra, Istvan Pócsi, Florentine Marx

**Affiliations:** 1 Biocenter, Division of Molecular Biology, Innsbruck Medical UniversityInnsbruck, Austria; 2 Sandoz GmbHKundl, Austria; 3 Department of Microbial Biotechnology and Cell Biology, Faculty of Science and Technology, University of DebrecenDebrecen, Hungary

**Keywords:** *Penicillium chrysogenum*, Antifungal protein PAF, Asexual development, Conidiation

## Abstract

*Penicillium chrysogenum* secretes a low molecular weight, cationic and cysteine-rich protein (PAF). It has growth inhibitory activity against the model organism *Aspergillus nidulans* and numerous zoo- and phytopathogenic fungi but shows only minimal conditional antifungal activity against the producing organism itself.

In this study we provide evidence for an additional function of PAF which is distinct from the antifungal activity against putative ecologically concurrent microorganisms. Our data indicate that PAF enhances conidiation in *P. chrysogenum* by modulating the expression of *brlA*, the central regulatory gene for mitospore development. A *paf* deletion strain showed a significant impairment of mitospore formation which sustains our hypothesis that PAF plays an important role in balancing asexual differentiation in *P. chrysogenum*.

## Introduction

The low molecular mass, cysteine-rich and cationic protein PAF from *Penicillium chrysogenum* exhibits cytotoxic activity towards a variety of filamentous fungi, among them zoo- and plantpathogens and the model organism *Aspergillus nidulans* [5, 13, 17, 23, 24]. The producing organism itself exhibits only slight conditional sensitivity towards PAF [17]. Antimicrobial cysteine-rich and cationic proteins like PAF are widely distributed in nature and represent a first line of defense against invading microorganisms in eukaryotes [4, 12, 21]. Some of the best characterized antimicrobial proteins are the defensins of plants [2, 46]. Plant defensins were shown to be systemically induced upon fungal infection in the vegetative tissues of various plant species [7, 11, 35, 45]. In contrast, the function of antimicrobial proteins from prokaryotes and lower eukaryotes is less well studied.

The benefit of the expression of antifungal proteins in ascomycetes, for example, could be an ecological advantage for the producing organisms in the competition for nutrients [23, 26], similarly to the function of fungal secondary metabolites as reported by [37]. This would imply the inducibility of the expression of antifungal proteins in the presence of microbial competitors or under unfavourable growth conditions.

The ascomycete *Aspergillus giganteus* expresses the PAF homologous antifungal protein AFP [25, 53]. Co-cultivation studies of *A. giganteus* with various AFP-sensitive and resistant microorganisms revealed that induction of *afp* expression was primarily dependent on the culture conditions (alkaline pH, carbon starvation, heat-shock, presence of excess NaCl and ethanol), but to a lesser extent on the presence of co-cultivants [27]. Similarly, we found no evidence that the production of PAF can be induced by the co-cultivation with other molds (unpublished data). Therefore, it is more likely that environmental stimuli play a major role in gene induction [23, 27]. Although the 5′-upstream region of the *paf* gene carries several putative regulatory elements that might be involved in the transcriptional regulation of the gene in response to environmental signals [23] the *paf* expression profile in *P. chrysogenum* does not parallel that of *afp* in *A. giganteus* [28]. Until now the significance of PAF production in *P. chrysogenum* cultures remained unclear and led us to hypothesize that PAF might exert an additional function, possibly the modulation of asexual development. Our assumption based upon the observation that PAF accumulates in the supernatant of *P. chrysogenum* liquid cultures in the stationary growth phase (72–96 h) [23] and that transcription of the *paf* orthologous gene *afp* occurs in *A. giganteus* surface cultures when aerial hyphae form [28].

In this study we show that *paf* mRNA accumulated in a time dependent manner in *P. chrysogenum* surface cultures which correlated with the expression of the conidiophore-specific regulator gene *brlA* and the onset of conidiation. Deletion of *paf* repressed *brlA* and the developmentally regulated genes *rodA* and *rodB* and resulted in a significant reduction of the conidiospore number. Thus, for the first time, we provide evidence that the antifungal protein PAF covers an important role as signaling molecule in the mitospore development of *P. chrysogenum*.

## Materials and methods

### Strains and growth conditions

*P. chrysogenum* Q176 wild-type (ATCC 10002) was grown on minimal medium (MM) containing per litre: 3 g NaNO_3_, 0.5 g KCI, 0.5 g MgSO_4_ · 7 H_2_O, 0.1 g FeSO_4_ · 7 H_2_O and 2% sucrose in 25 mM K-phosphate buffer (pH 5.8) In the case of the *P. chrysogenum* Δ*brlA* mutant (Sandoz GmbH strain collection, Kundl, Austria) and its recipient strain Δ*Pc*ku70 [16] 2.5 g arginine was added to MM. All surface cultures used in this study were synchronized, unless otherwise stated. To synchronize surface cultures, approx. 6 × 10^8^–10^9^ spores were grown at 25 °C for 19 h in 200 ml MM. The Δ*Pc*ku70 and Δ*brlA* strains, however were cultivated longer (36 h) because of lower proliferation rates. Then the mycelia were harvested by filtration and transferred to solid MM, and were further incubated for various cultivation times. Alternatively, 10^5^ conidia were point inoculated onto solid MM and conidiospores were harvested after various cultivation times.

### Determination of conidial counts

The colony diameter of point inoculated *P. chrysogenum* surface cultures was determined before the conidia were harvested. From synchronized surface cultures a defined area (8 mm diameter) was cut out. Conidia were harvested by vortexing the excised surface culture in spore suspension (0.9% NaCl and 0.01% tween), conida were counted and the counts were divided by the colony area to obtain the number of conidiospores/cm^2^. Conidial yield data are means of three independent surface cultures. Statistical analysis was performed by using Microsoft Excel.

### PAF purification

PAF was purified from the supernatant of 72 h cultures of *P. chrysogenum* Q176. The supernatant was cleared by centrifugation and ultrafiltration and then loaded on a CM-sepharose column as described previously [17]. Eluted fractions containing PAF were pooled, dialyzed against phosphate buffer (10 mM Na-phosphate, 25 mM NaCl, pH 6.6), concentrated and filter sterilized. The protein concentration was determined photometrically and by SDS-PAGE.

### Northern analysis

Total RNA was isolated with TRI Reagent (Sigma-Aldrich) from *P. chrysogenum* surface culture and from purified conidia. Conidia were separated from the mycelia by filtration with nylon Cell Strainer (40 μm) (BD Biosciences), then concentrated by centrifugation and immediately used for RNA isolation. Ten micrograms of total RNA were fractionated on 1.2% formaldehyde–agarose gels, blotted onto Hybond-N membranes (Amersham Biosciences), and hybridized with digoxigenin-labeled probes (Boehringer Mannheim). Hybridization probes were generated by PCR amplification using the oligonucleotides opaf1 and opafrev for *paf* and obrlAfw and obrlArev for *brlA* (according to the annotated gene **AM920421**). Two genes are annotated in the *P. chrysogenum* genome with strong similarity to *A. nidulans rodA*. For PCR amplification we used orodAfw and orodArev for *rodA*, and orodBfw and orodBrev for *rodB* (according to the annotated genes **AM920437** and **AM920436**, respectively) (Table 1). All oligonucleotides were purchased from Microsynth.

**Table 1:** Oligonucleotides used in this study

oligo	sequence (5′ to 3′)
opaf1	GGTACCATCGCCCAAATCACCACAGTTG
opafrev	GATCGGATCCCTAGTCACAATCGACAGC
obrlAfw	TCCTACTCCCACGCCTAC
obrlArev	CCTGGCTCCTTGCACTTG
orodAfw	CTTACGCTCTTCCCCCTG
orodArev	GCTGGAAGGAGAGTTCTGG
orodBfw	ATGCAGTTCACTCTCTCCG
orodBrev	ACGAGGTCGTTGTTGGCC
opaf5	CGAAAAGGCAAAGGCAC
o5pafA1	CGATGCTACGTCACTTGTTAGCG
o5pafArev	ACGTGGATCCTATGAAGGGCTTGAGATGATG
o3pafAse	ACGTGTCGACATGGTCTCTGCGATCACCAGG
o3pafA2	CACAACCTTACGCATGCGGAG
o3pafArev	ACGTTCTAGACCAAAAGGCTTCCCCGTCATC
o5pafAse	ACGTGGTACCGACAGCTTAGTGGACCGGCAG
o5pafcomp	GATGGTACCACTTGCGTAATAACCGGG
o3pafcomp	CACGGTACCCTTCCTTGACTTACTCCC
onat1	CGCCGGTACGCGTGGATCGC
onat2	AGGCACTGGATGGGTCCTTCAC

### Fungal transformation, targeted gene disruption and genetic complementation

**Figure 1:**
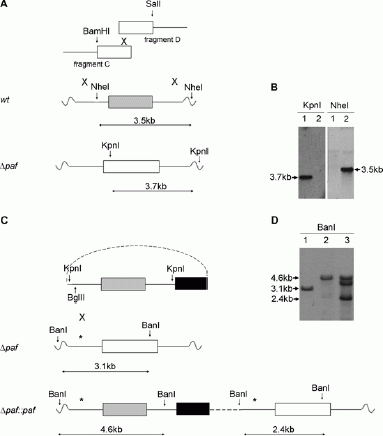
Generation and verification of the *P. chrysogenum* Δ*paf* (A, B) and Δ*pat::paf* (C, D) strains. (A, C) The white, grey and black boxes represent the nourseothricin-acetyltransferase gene *(nat1)*, the *paf* gene and the pyrithiamine resistance gene *(ptrA)*, respectively. The continuous lines indicate 2.1 kb and 2.2 kb of the 5′-UTR and 3′-UTR of the *paf* gene, respectively. The crosses show regions involved in homologous recombination. The dashed line represents the plasmid backbone. Restriction sites used for cloning and Southern blot analysis are indicated by arrows and the predicted fragments detectable by Southern blot analysis are marked by double arrows. The position of the 5′-UTR-specific digoxigenin probe is indicated by an asterisk (*). Cloning was performed as described in Materials and Methods. (B) Southern blot hybridization of KpnI- and NheI-digested genomic DNA hybridized with a *nat1*-specific and a *paf*-specific digoxigenine probe, respectively. (D) Southern blot hybridization of BanI-digested genomic DNA hybridized with a *paf* 5′-UTR-specific digoxigenine probe. (B) and (D) Lane 1: Δ*paf*, lane 2: wild-type, lane 3: Δ*pat::paf*.

Homologous recombination occurs very rarely in *P. chrysogenum*. Therefore, the bipartite marker technique was used for generating a Δ*paf* mutant strain [32]. *P. chrysogenum* wild-type was co-transformed with two PCR constructs, each containing an incomplete fragment of the nourseothricin-acetyltransferase gene *(nat1)* [19] fused to 2.1 kb and 2.2 kb of the 5′-UTR and 3′-UTR of *paf* respectively. In brief, each flanking region was amplified from wild-type genomic DNA using primer o5pafA1 and o5pafArev for the 5′-UTR (fragment A, 2.1 kb), and o3pafAse and o3pafA2 for the 3′-UTR (fragment B, 2.2 kb). Subsequent to gel-purification, the fragments were digested with *BamH*I and *Sal*I, respectively. The *nat1* selection marker was released from plasmid pD-NAT1 (a kind gift from Ulrich Kück, Bochum, Germany) by digestion with *BamH*I and *Sal*I, and ligated to the fragments A and B. For generation of Δ*paf*, two overlapping PCR fragments were amplified from the respective ligation products using primers o5pafAse and onat1 for fragment C (2.8 kb) and primers onat2 and o3pafArev for fragment D (2.4 kb). The PCR fragments C and D shared a 400 bp overlap within the *nat1* cassette, which served as a potential recombination site during transformation (Fig. 1A and Table 1). Subsequently, *P. chrysogenum* Q176 was co-transformed with the overlapping fragments C and D. Protoplastation was performed according to the modified protocol of [8] and [18]. Briefly, a 48 h *P. chrysogenum* liquid culture was harvested by filtration and washed with sterile water. The digestion of the fungal cell wall was accomplished with 300 mg Glucanex (Novozymes, Denmark) in 15 ml lysis solution (0.7 M KCl, in 50 mM K-phosphate buffer, pH 5.8) per 2 g semidry mycelium for 3 h by gentle shaking. Protoplasts were filtered through folded filter paper (595½, Schleicher & Schuell, Germany), washed with 0.7 M KCl and resuspended in KCM solution (per litre: 52.2 g KCl, 8 g CaCl_2_, 2 g MOPS, pH 5.8). The transformation was carried out as described previously [47] using 10 μg DNA. Homologous integration of each fragment into the genome at the *paf* locus allowed recombination of the incomplete *nat1* fragments and generation of an intact resistance gene against nourseothricin at the site of recombination. Transformants were selected on solid MM supplemented with 200 μg/ml nourseothricin (Jena Bioscience, Germany). Accurate gene deletion was confirmed by Southern hybridization (Fig. 1B). Hybridization probes were generated by PCR amplification using oligonucleotides opaf1 and opafrev for the *paf* probe and onat1 and onat2 for the *nat1* probe (Table 1).

For reintegration of the *paf* gene into the Δ*paf* strain, the plasmid pSK275 was used, which contains the am-picillin resistance gene for propagation in *E. coli* and the pyrithiamine resistance gene for selection of transformed *P. chrysogenum*. The *P. chrysogenum* genomic DNA (4400 bp), containg the *paf* gene (422 bp) and approx. 2050 bp of the 5′-UTR and 1950 bp of the 3′-UTR, was PCR amplified using primer o5pafcomp and o3pafcomp, each containing an additional *KpnI* restriction site (Table 1). The amplified PCR fragment was gelpurified and ligated into pSK275. Fifteen μg plasmid was linearized with BglII and transformed into protoplasts of the Δ*paf* strain as described above (Fig. 1C). Transformants were single spored on pyrithiamine hydrobromide (0.6 μg/ml) containing MM agar plates. The reintegration of the reconstitution cassette into the deletion mutant was proved by Southern-blot analysis by using a 5′-UTR specific hybridization probe generated by PCR amplification with the oligonucleotides o5pafcomp and opaf5.

## Results

### The expression of the *paf* gene is temporally and spatially regulated during asexual development

**Figure 2:**
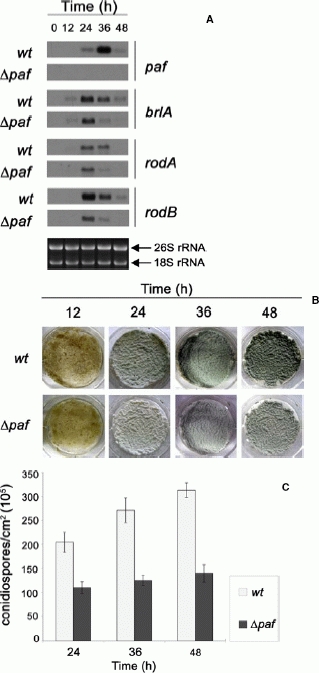
Deletion of the *paf* gene negatively interferes with the expression of *brlA, rodA* and *rodB* and represses mitospore development in *P. chrysogenum*. A Northern blot analysis of *paf, brlA, rodA* and *rodB* expression in *P. chrysogenum* wild-type and Δ*paf* mutant strain. Total RNA was extracted from surface culture after 0, 12, 24, 36 and 48 h of exposure to air and cultivation on solid MM. Ten μg of total RNA were loaded into each well and hybridized with digoxigenin probes specific for the respective mRNAs. Ethidium-bromide-stained 26S and 18S rRNA was used as a loading control. **B** Synchronized surface cultures were photographed at 12, 24, 36 and 48 h after the exposure of mycelia to air. **C** The number of conidiospores (×10^5^) of 24, 36 and 48 h cultures is given in conidiospores/cm^2^.

A time course experiment revealed that *paf* mRNA was detectable in *P. chrysogenum* wild-type surface cultures starting from 24 h after synchronization. The expression reached a maximum at 36 h before it decreased again (Fig. 2A). This expression pattern correlated with the expression of the central regulator for asexual development, *brlA*, with the transcription of the developmentally regulated genes *rodA* and *rodB* and with the mitospore production (Fig. 2 A, B). However, *brlA, rodA* and *rodB* transcription preceeded that of *paf* (Fig. 2A).

**Figure 3:**
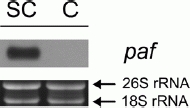
Northern blot analysis of the expression of *paf* in *P. chrysogenum* hyphae and conidiospores. Total RNA of a 36 h *P. chrysogenum* wild-type surface culture (SC) and from purified conidia (C) was extracted. Ten μg of total RNA were loaded into each well, blottetd and hybridized with a *paf* specific digoxigenin probe. Ethidiumbromide-stained 26S and 18S rRNA is shown as loading control.

Northern blot analysis from a 36 h old *P. chrysogenum* wild-type surface culture and from purified conidia indicated that *paf* expression was spacially distributed. The expression pattern revealed that the *paf* gene was not transcribed in conidia but in the other parts of the surface culture which contain hyphae and conidio-phores (Fig. 3).

### Deletion of *paf* reduces conidiation in *P. chrysogenum*

To further analyze the function of PAF in the developmental process of *P. chrysogenum* we deleted the *paf* gene and replaced it by the nourseothricin-acetyltransferase gene *nat1* which confers nourseothricin-resistance to the transformants [19]. The *paf* gene replacement by *nat1* was proved by Southern hybridization (Fig. 1B). To test whether deletion of *paf* affects conidiation, the Δ*paf* strain and the wild-type strain were grown on MM plates and the total conidial number was determined after 48 h of incubation (Table 2). The Δ*paf* mutants generated ∼2.3 × 10^7^ ± 1.3 × 10^6^ conidia/cm^2^ compared to ∼4.9 × 10^7^ ± 4.5 × 10^6^ conidia/cm^2^ of the wild-type strain. This corresponds to 53% attenuation in mutant strain compared to the control. Reduction of conidiation was even more prominent after 6 days of cultivation: ∼2.1 × 10^7^ ± 6.0 × 10^5^ conidia/cm^2^ in the mutant compared to ∼7.0 × 10^7^ ± 7.9 × 10^6^ conidia/cm^2^ in the wild-type which corresponds to a decreased conidiation of 70% in Δ*paf*. Importantly, no effects on the vegetative growth, hyphal morphology or germination efficiency were detected in Δ*paf* (data not shown).

**Table 2:** The conidial number of *P. chrysogenum* wild-type and Δ*paf* that were point inoculated (10^5^ conidia) on solid MM agar plates

incubation time	number of conidia/cm^2^ (% of relative change in conidiation efficiency)^a^	
	
	wt	Δ*paf*
48 h	4.9 × 10^7^ ± 4.5 × 10^6^	2.3 × 10^7^ ± 1.3 × 10^6^ (−53%)
6 d	7.0 × 10^7^ ± 7.9 × 10^6^	2.1 × 10^7^ ± 6.0 × 10^5^ (−70%)

a The percentage (%) of the relative change in conidiation efficiency of the mutants compared to the wild-type strain (= 100%) is indicated in brackets.

In a next step, we characterized the conidiation defect in more detail and performed time course experiments with synchronized surface cultures of the Δ*paf* and the wild-type strain. The number of conidia was significantly reduced in the Δ*paf* mutant compared to the wild-type (Fig. 2C; Table 3). The defect became most evident 48 h after exposition of the mycelium to air. At this time point the wild-type strain produced ∼3.1 × 10^7^ ± 1.5 × 10^6^ conidia/cm^2^ and Δ*paf* only ∼1.4 × 10^7^ ± 1.8 × 10^6^ conidia/cm^2^ which reflects a 55% decrease in conidiation compared to the wild-type (Table 3).

**Table 3:** The conidial number of a synchronized culture of *P. chrysogenum* wild-type and the Δ*paf* mutant

incubation time	number of conidia/cm^2^ (% of relative change in conidiation efficiency)^a^	
	
	wt	Δ*paf*
12 h^b^	n.d.	n.d.
24 h	2.1 × 10^7^ ± 2.1 × 10^6^	1.1 × 10^7^ ± 1.3 × 10^6^ (−48%)
36 h	2.7 × 10^7^ ± 2.6 × 10^6^	1.3 × 10^7^ ± 1.1 × 10^6^ (−52%)
48 h	3.1 × 10^7^ ± 1.5 × 10^6^	1.4 × 10^7^ ± 1.8 × 10^6^ (−55%)

a The percentage (%) of the relative change in conidiation efficiency of the mutant compared to the wild-type strain (= 100%) is indicated in brackets.

b No conidiation was observed after 12 h of exposure of the preculture to the air. Therefore the number of conidia was not determined (n.d.) at this early time point.

The transcriptional analysis of the developmentally expressed genes *brlA, rodA* and *rodB* supported the observed phenotype. The transcription of these genes was repressed in the Δ*paf* strain. In detail, in the mutant strain less mRNA of *brlA, rodA* and *rodB* was detectable and the period of transcription was shorter than in the control (Fig. 2A). This indicated that PAF indeed modulates the asexual development on transcriptional level in *P. chrysogenum*.

### Complementation of Δ*paf* restores mitospore development

Retransformation of the *paf* wild-type copy resulted in pyrithiamine resistant clones with site-specific and additional ectopic integrations of the transforming cassette (Fig. 1D). The complemented strains secreted PAF into the supernatant after 72 h of submers culture as observed by SDS-PAGE (data not shown) and 48 h old synchronized surface cultures of Δ*paf::paf* showed restored conidial development: the conidial counts were ∼2.9 × 10^7^ ± 2.1 × 10^6^ conidia/cm^2^ in the complemented strain compared to ∼3.2 × 10^7^ ± 1.6 × 10^6^ in the wild-type.

Since PAF is a secreted protein, we also attempted to restore the conidiation defficiency by exposing the *P. chrysogenum* Δ*paf* mutant to purified PAF protein in agar diffusion assays. However, no increase of the conidiation could be observed at the conditions tested (data not shown).

### The expression of *paf* is not regulated by *brlA*

**Figure 4:**
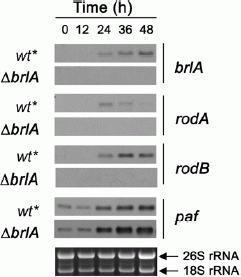
Northern blot analysis of *brlA, rodA, rodB* and *paf* expression in a *P. chrysogenum* Δ*brlA* mutant. Total RNA was isolated from surface culture of the recipient strain Δ*Pcku70* which is designated as wt* and the Δ*brlA* strain after 0, 12, 24, 36, and 48 h of cultivation on solid MM. Importantly, synchronization started from a 36 h preculture of both strains. Ten μg of total RNA were loaded in each well, blotted and hybridized with digoxigenin probes specific for the respective gene transcripts. Ethidiumbromide-stained 26S and 18S rRNA was used as a loading control.

Generally, genes under the control of *BrlA* contain *BrlA* response elements (5′-(C/A)(G/A)AGGG(G/A)-3′) in their promoter regions [10]. *In silico* analysis of the *paf* 5′-UTR revealed 2 putative *BrlA* response elements (5′-CAAGGG-3′ at −784 bp and 5′-AAAGGG-3′ at −1138 bp from the start codon, respectively) in the *paf* promoter region. Since we could show in this study that PAF modulates the asexual differentiation of *P. chrysogenum*, the question arised if *paf* gene expression is regulated by a *BrlA*-dependent mechanism. To this end we tested the *paf* transcription profile in a *P. chrysogenum* Δ*brlA* mutant (fungal strain collection of Sandoz GmbH, Kundl, Austria [40]. The Δ*brlA* mutant was generated using a *Pcku70* deletion strain with an improved gene targeting efficiency [16]. The Δ*brlA* deletion strain revealed a similar phenotype as described in *A. nidulans*, namely a severe defect in conidiation (data not shown) and a repression of *rodA* and *rodB* expression (Fig. 4). We verified that *paf* and *brlA* expression correlated in the recipient strain Δ*Pcku70* (Fig. 4). It is important to note here that the expression pattern of both genes in Δ*Pcku70* slightly differed from the wild-type strain Q176 (Fig. 2A). This could be explained by the fact that the Δ*Pcku70* and Δ*brlA* mutant strains had significantly lower proliferation rates when grown under the experimental conditions applied in this study. Therefore, we had to use older precultures (36 h instead of 19 h) to start synchronization in this experiment. Under these conditions, *paf* is already transcribed in both precultures as it is also true for a 36 h liquid culture of the wild-type strain Q176 (data not shown).

However, Northern blot analysis with the Δ*brlA* mutant indicated that *paf* gene transcription was not affected by the deletion of *brlA*, but resembled the gene expression pattern of the recipient strain Δ*Pcku70* (Fig. 4). Importantly, *paf* transcription in Δ*Pcku70* is similar to that in the parental strain P2niaD18, which excludes an effect of *ku70* gene deletion on *paf* expression (Table 4). Therefore, *paf* seems not to be under *BrlA* regulation.

**Table 4:** Fold change in *paf* expression in the *P. chrysogenum* reference strain P2niaD18 and the mutant strains Δ*PcvelA* and Δ*PclaeA* compared to the recipient strain Δ*Pcku70* after 48 h, 60 h and 96 h of cultivation. Values were calculated according to the microarray data published by [15] (NCBI Gene Expression Omnibus (GEO), accession number GSE18585)

strain	48 h	60 h	96 h
P2niaD18	0.4	0.8	−1.0
Δ*PcvelA*	−15.2	−28.6	−10.2
Δ*PclaeA*	0.4	0.8	1.0

## Discussion

In this study, we provide evidence that the *paf* gene product is involved in the regulation of asexual development in *P. chrysogenum*. Conidiation is best studied in *Aspergillus* sp. The central regulator in asexual development is BrlA which activates the specific gene expression at the beginning of conidiophore vesicle formation [29]. Other factors that are closely connected to mito-spore development are the low molecular weight hydrophobic proteins RodA and RodB which form highly insoluble complexes in the outer layers of the fungal cell wall [54]. Whereas RodA forms the conidial rodlet layer, RodB is not required for rodlet formation but seems to play a role in the building of the conidial cell wall [33]. Hydrophobins are BrlA-regulated and developmentally expressed [10]. They were attributed protective functions such as water repellence, protection against desiccation, resistance to killing by alveolar macrophages, high resistance to solubilisation and chemical degradation [33, 34, 43]. So far, these genes have not been characterized in detail in *P. chrysogenum*. However, since these genes are higly conserved within filamentous ascomycetes [50], a conserved function can be attributed to the *P. chrysogenum* genes as well.

Therefore, we used in our study *brlA* and *rodA/rodB* as marker genes to investigate the PAF-dependent regulation of conidiation in *P. chryosgenum*. We examined the expression profile of *paf, brlA, rodA* and *rodB* in *P. chryosgenum* surface cultures and found all four genes simultaneously expressed. Furthermore, the accumulation of the respective gene transcripts correlated with the onset of conidiation. This gene expression pattern and conidiation were significantly reduced in a *paf* deletion strain. Notably, unlike the repression of both hydrophobin encoding genes *rodA* and *rodB* in the *P. chrysogenum brlA* deletion mutant, the regulation of *paf* occured independently from BrlA. Based on our finding we propose the following tentative model which, however, needs to be tested in further experiments: PAF influences asexual development by indirectly modulating *brlA* expression. This could occur for example by varying the activity of AbaA, StuA or protein X, which are modulators of *brlA* expression (AbaA, StuA, X) or BrlA activity (X) [1].

Unexpectedly, we were not able to restore the wild-type phenotype of the Δ*paf* strain by external administration of purified PAF protein. Possible explanations for this result could be: (i) the extremely fine tuning of developmental processes in fungi which depend on environmental conditions, cell cycle, nutritional stages, age of the colony, activation of signaling cascades etc. In this respect, the simple addition of PAF to the growth medium seems not to be effective, at least in the experimental setup that we used so far, as its activity might strongly depend on the overall physiological condition of the fungal cells. (ii) Another possibility could be the redox-state of the PAF protein under the applied assay conditions. PAF contains six cysteine residues forming three disulfide bonds – a perfect feature for oxidative or reductive protein transformation [6]. A conformational change taking place during secretion or upon contact with molecular structures/receptors on the fungal cell surface could influence/modulate the activity of PAF, as proposed for conidiogenol – a precursor of the development modulating conidiogenone. This diterpene requires oxidative transformation into an active form and conidiation induction likely takes place *via* a specific cellular receptor [38, 39]. Thus, the activation by the change of the redox state could also account for the activity and the variable function of PAF [6]. However, structural investigations are underway to clarify this assumption.

Notably, (i) and (ii) might not necessarily exclude each other, but could together explain our observation, (iii) Finally, the secrection process of PAF *per se* might have regulatory potential as well. The premature anti-fungal protein contains in addition to the signal sequence an N-terminal prosequence which is cleaved off when the protein is secreted. This prosequence was attributed an intramolecular chaperone function [24]. However, it cannot be excluded, that the prosequence itself or the maturation of PAF might elicit a signal. Importantly, our assumption that PAF plays a role in development was further corroborated by the report of Meyer et al. that the expression of the orthologous *A. giganteus afp* gene is under strict regulation by distinct environmental stimuli and specific developmental stages, pointing towards an AFP function only under very defined physiological conditions [28].

Most interestingly, when we finalized this manuscript a genome wide expression study of the global regulator for development and secondary metabolism *PcvelA* and the central regulator for secondary metabolism *PclaeA* in *P. chrysogenum* became available [15]. The microarray data indicate a repression of *paf* in the Δ*PcvelA* mutant, but no change of *paf* expression in the Δ*PcleaA* mutant (Table 4). This further corroborates our data that *paf* is developmentally regulated. However, this relation awaits detailed investigation in the near future.

The molecular mechanism governing the induction of conidiation in filamentous fungi has been intensively studied in recent years uncovering different steps of signalling pathways, mainly in the model organisms *A. nidulans* and *Neurospora crassa* [1, 42]. Nevertheless, the question of the conidiation inducing signals remained partly unresolved. Apart from the emergence of hyphae to the air [31], nutrient starvation [41], light [30], high osmolarity [3, 52], and chemical signals [50] are recognised to be the crucial stimuli for this process. Notably, endogenous extracellular molecules can trigger conida-tion and/or modulate the ratio of asexual and sexual development in fungi as well [14, 31]. For example, an as yet unidentified *fluG* gene dependent extracellular factor has been proposed to exist in *A. nidulans*, which is involved in conidiation induction [1, 20]. Fungal oxylipins (hormone-like psi factors) regulate asexual and sexual development [9, 48, 49], and the discovery of the conidiation inducing molecule conidiogenone in *Penicillium cyclopium* [38, 39, 51] point to the possibility that autoinducer-mediated mechanisms are widespread among filamentous fungi.

Based on our findings, we can draw some conditional parallels between the effect of PAF and other components that modulate development. Oxylipins exhibit pleiotropic effects by activating a wide range of cellular responses – apart from their role in regulating mito-and meiospore development. Similarly to the antifungal activity found in PAF, oxylipins also elicit defence and stress responses and impair the mycelial growth and spore germination of various plant-pathogens [36, 49].

The variation in the mode of action of PAF could reside in its ability to induce different signalling pathways [22–24]. This might rely on the existence of multiple receptors which exert distinct responses in different tissues and organisms. Indeed, PAF does not augment the conidiation efficiency, but inhibits hyphal elongation and conidiation in other filamentous fungi [5, 13, 17, 23].

In conlusion, we propose that PAF might act in a similar way to quorum sensing molecules which direct distinct cellular responses to environmental stimuli [39, 44, 49]. Our study provide evidence that PAF might help to adjust to variable environmental conditions by balancing asexual spore development *via brlA* regulation in *P. chrysogenum*. At the same time, PAF transmits a growth inhibition signal in fungal organisms that have been categorized so far as "PAF-sensitive". This effect in combination with a highly efficient propagation of conidia undoubtly provides a fitness mechanism to *P. chrysogenum* and an ecological advantage over concurring organisms. The existance of different sets of receptors on the fungal cell surface, a variation in the redox state of PAF and/or a modulation in the transmission of the signal might provide an explanation for these pleiotropic effects of PAF.
